# Mining and evaluation of adverse event signals for capmatinib based on the FAERS database

**DOI:** 10.3389/fphar.2024.1417661

**Published:** 2024-09-24

**Authors:** Xinnan Chen, Ying Jiang, Haohao Zhu, Man Tian

**Affiliations:** ^1^ School of Pediatrics, Nanjing Medical University, Nanjing, China; ^2^ Mental Health Center of Jiangnan University, Wuxi Central Rehabilitation Hospital, Wuxi, China; ^3^ Department of Respiratory Medicine, Children’s Hospital of Nanjing Medical University, Nanjing, China

**Keywords:** Capmatinib, adverse events, FAERS database, ear and labyrinth disorders, renal and urinary disorders

## Abstract

**Objective:**

To conduct a comprehensive data analysis based on the FDA’s Adverse Event Reporting System (FAERS) to mine possible adverse event (AE) signals of Capmatinib, providing valuable references for its clinical application.

**Methods:**

Capmatinib was the primary suspected drug in the search of FAERS database from the second quarter of 2020 to the fourth quarter of 2023. Data processing, screening, and classification were performed using methods such as the Reporting Odds Ratio (ROR), Proportional Reporting Ratio (PRR), Bayesian Confidence Propagation Neural Network (BCPNN), and Multi-item Gamma Poisson Shrinker (MGPS).

**Results:**

A total of 1,991 AE reports directly related to Capmatinib were screened, identifying 269 Preferred Terms (PTs) involving 26 System Organ Classes (SOCs). Besides the AEs recorded in the drug label (such as edema, nausea, fatigue, and dyspnea), the study unearthed other high-risk AEs not listed in the label, including Renal and urinary disorders, Vocal cord paralysis, and Ear and labyrinth disorders. Among these, renal and urinary disorders, and ear and labyrinth disorders had a higher frequency and intensity of signals, suggesting that their mechanisms of occurrence could be a future research direction.

**Conclusion:**

This study uncovered new potential AEs of Capmatinib based on the FAERS database, providing reference for its safe clinical use. Special attention should be given to the occurrence of ear and labyrinth disorders and renal and urinary disorders, primarily presenting as pseudo-acute kidney injury, during treatment.

## 1 Introduction

Lung cancer is one of the most common respiratory malignancies worldwide, with high incidence and mortality rates (Herbst et al., 2018). There are two main types: non-small cell lung cancer (NSCLC), which accounts for 85%, and small cell lung cancer, accounting for 15% (Duma et al., 2019). Based on histopathological characteristics, NSCLC can be further classified into adenocarcinoma and squamous cell carcinoma (Travis et al., 2015). NSCLC has the highest incidence among lung cancers, and its incidence continues to rise with the deterioration of air quality, posing a significant threat to human life and health (Chu et al., 2023). In recent years, with the advent of the era of precision oncology, the field of drug therapy for NSCLC has rapidly developed, and more clinical drug research involving different stages and methods of treatment has achieved remarkable results. The mesenchymal-epithelial transition (MET) proto-oncogene is associated with various tumors, including NSCLC. MET exon 14 skipping mutations and amplification are the two most prominent manifestations of MET pathway activation (Hsu et al., 2023a). Capmatinib, as a selective MET receptor inhibitor, can block MET-dependent cancer cell proliferation and survival by inhibiting MET exon 14 skipping mutations, achieving an anti-tumor effect. Most of its adverse events (AEs) are grade 1 or 2, predictable, and can be managed with dose adjustment, thanks to its availability in oral tablet form (Valencia Soto et al., 2023). Therefore, Capmatinib has a promising clinical application prospect.

In a phase II study involving 364 patients across multiple cohorts, Capmatinib’s therapeutic effect on MET dysregulated advanced NSCLC patients was evaluated. The data showed that in patients with advanced NSCLC with MET exon 14 skipping mutations, 41% (95% CI, 29–53) of previously treated patients showed overall response, while 68% (95% CI, 48–84) of treatment-naïve patients showed overall response. Limited efficacy (7%–12% overall response) was observed in previously treated patients with MET gene copy number amplification less than 10. In patients with MET gene copy number amplification of 10 or more, 29% (95% CI, 19–41) of previously treated patients and 40% (95% CI, 16–68) of treatment-naïve patients showed overall response. These results indicate that Capmatinib has significant efficacy, particularly in treatment-naïve patients with NSCLC with MET exon 14 skipping mutations. For patients with high MET gene copy number amplification, Capmatinib also demonstrated some anti-tumor effects (Wolf et al., 2020). Most of Capmatinib’s safety AEs are reversible by adjusting the dose, with common adverse reactions including peripheral edema, musculoskeletal pain, nausea, and vomiting, which are relatively mild and have minimal impact on the cardiovascular and central nervous systems (Heist et al., 2021). However, as research and clinical trials progress, Capmatinib has also been reported to possibly cause adverse reactions such as interstitial lung disease, toxic hepatitis, and pancreatitis, which can significantly affect patients’ efficacy and quality of life (Valencia Soto et al., 2023). Therefore, in clinical practice, further evaluation of the drug’s safety is necessary.

Given the relatively short time since Capmatinib’s market approval, there is currently a lack of comprehensive assessment of its adverse reactions. Although some possible AEs of Capmatinib have been identified through trials and clinical case reports, the results are often limited due to factors such as time, experimental conditions, and the scale of the subjects. Therefore, mining real-world data can effectively complement the deficiencies of early experimental findings. The FDA Adverse Event Reporting System (FAERS) collects and monitors a large number of drug AEs (Alatawi and Hansen, 2017). To more comprehensively and deeply mine the safety and AEs of Capmatinib in the real world, this study aims to conduct an in-depth analysis of the FAERS database to evaluate potential AE signals of Capmatinib, providing references for patients and physicians in clinical use of the drug.

## 2 Methods

### 2.1 Data source

The data used in this study were obtained from the FAERS database (https://www.fda.gov/drugs/development-approval-process-drugs/drug-approvals-and-databases). This database, based on a spontaneous reporting system from patients and healthcare professionals, has accumulated a substantial amount of real-world data on drug AEs, which is one of the largest drug AE monitoring databases globally, providing a large quantity of data resources for drug safety studying. The study extracted data from the second quarter of 2020 to the fourth quarter of 2023.

### 2.2 Data extraction

The search was conducted using the drug name “Tabrecta” and the commercial name “Capmatinib” as keywords, to retrieve AE reports for Capmatinib as the primary suspected drug from the second quarter of 2020 to the fourth quarter of 2023 on 5 February 2024. AE reports were classified into system organ classes (SOCs) using the preferred terms (PTs) in the Medical Dictionary for Regulatory Activities (MedDRA 26.0) (Rothman et al., 2004). Cases with more than three records in the FAERS database were selected, and reports with missing patient information or duplicates were excluded to obtain the AE report information related to Capmatinib.

### 2.3 Data analysis

Descriptive analysis was used to present the characteristics of all AE reports related to Capmatinib. This study employed descriptive statistical methods such as the Reporting Odds Ratio (ROR), Proportional Reporting Ratio (PRR), Bayesian Confidence Propagation Neural Network (BCPNN), and Multi-item Gamma Poisson Shrinker (MGPS) to conduct a comprehensive statistical analysis of the data (Evans et al., 2001; Bate et al., 1998; DuMouchel, 1999; Brown, 2004). The ROR and PRR methods can identify abnormally high reporting ratios of adverse events, highlighting the risk of Capmatinib adverse event signals. The BCPNN, as a more advanced algorithm, can capture potential drug-adverse event associations with higher reliability. The MGPS algorithm is more comprehensive, as it quantifies adverse event signals by considering the number of reports and background risk. By mining Capmatinib’s AEs from different perspectives and aspects, the study aimed to achieve more credible and persuasive drug AE assessment results. Specific calculation formulas can be found in Tables 1, 2.

**TABLE 1:** Four grid table.

	Target AEs	Non-target AEs	Total
Capmatinib	a	b	a+b
Non- Capmatinib	c	d	c + d
Total	a+c	b + d	N = a+b + c + d

**TABLE 2:** ROR, PRR, BCPNN, and EBGM methods, formulas, and thresholds.

Method	Formula	Threshold
ROR	$\text{ROR}=\frac{\left(a/c\right)}{\left(b/d\right)}=\frac{ad}{bc}$ $\text{SE}\left(\text{lnROR}\right)=\sqrt{\left(\frac{1}{a}+\frac{1}{b}+\frac{1}{c}+\frac{1}{d}\right)\;}$ $95\%\text{CI}=e^{\ln\left(\text{ROR}\right)\pm 1.96\sqrt{\;\left(\frac{1}{a}+\frac{1}{b}+\frac{1}{c}+\frac{1}{d}\right)\;}}$	a ≥3 and 95% CI (lower limit) > 1
PRR	$\text{PRR}=\frac{a/\left(a+b\right)}{c/\left(c+d\right)}$ SE (lnPRR) = $\sqrt{\;\frac{1}{a}-\frac{1}{a+b}+\frac{1}{c}-\frac{1}{c+d}\;}$ 95%CI = $e^{\ln\;\left(\text{PRR}\right)\pm 1.96\sqrt{\;\frac{1}{a}-\frac{1}{a+b}+\frac{1}{c}-\frac{1}{c+d}\;}}$	a ≥3 and 95% CI (lower limit) > 1
BCPNN	IC = $\log_{2}\frac{p\left(x,y\right)}{p\left(x\right)p\left(y\right)}=\log_{2}\frac{a\left(a+b+c+d\right)}{\left(a+b\right)\left(a+c\right)}$ E (IC) = $\log_{2}\frac{\left(a+\gamma 11\right)\left(a+b+c+d+\alpha \right)\left(a+b+c+d+\beta \right)}{\left(a+b+c+d+\gamma \right)\left(a+b+\alpha 1\right)\left(a+c+\beta 1\right)}$ V(IC) = $\frac{1}{{\left(\ln2\right)}^{2}}\left\{\left[\frac{\left(a+b+c+d\right)-a+\gamma -\gamma 11}{\left(a+\gamma 11\right)\left(1+a+b+c+d+\gamma \right)}\right]+\left[\frac{\left(a+b+c+d\right)-\left(a+b\right)+\alpha -\alpha 1}{\left(a+b+\alpha 1\right)\left(1+a+b+c+d+\alpha \right)}\right]+\left[\frac{\left(a+b+c+d\right)-\left(a+c\right)+\beta -\beta 1}{\left(a+c+\beta 1\right)\left(1+a+b+c+d+\beta \right)}\right]\right\}$ $\gamma =\gamma 11\frac{\left(a+b+c+d+\alpha \right)\left(a+b+c+d+\beta \right)}{\left(a+b+\alpha 1\right)\left(a+c+\beta 1\right)}$ *IC-2SD = E(IC)-2* $\sqrt{V\left(IC\right)}$	IC025 > 0
EBGM	$\text{EBGM}=\frac{a\left(a+b+c+d\right)}{\left(a+c\right)\left(a+b\right)}$ $95\%\text{CI}=e^{\ln\left(\text{EBGM}\right)\pm 1.96\sqrt{\;\left(\frac{1}{a}+\frac{1}{b}+\frac{1}{c}+\frac{1}{d}\right)\;}}$	EBGM05 > 2

## 3 Results

### 3.1 Basic information of AE reports

In this study, after strict selection and analysis from the FAERS database, 1,991 AE reports directly related to Capmatinib from the second quarter of 2020 to the fourth quarter of 2023 were analyzed (Table 3). Among these reports, 49.37% were from female patients, slightly higher than the 39.73% from male patients. Notably, 53.59% of the reports were submitted by consumers, far exceeding those reported by pharmacists (13.46%). In terms of age distribution, the elderly population aged 65 and above accounted for the largest proportion at 23%. The year with the fewest reported AEs was 2020, particularly in the second quarter (0.70%), with reports from 2021 to 2023 remaining relatively stable, around 30%. In terms of report geography, the United States accounted for the majority, with 62.53%. Regarding severe AE outcomes, there were 518 death reports, accounting for 26.02% of the total. In addition, there were 313 reports of hospitalization or extended hospital stays, accounting for 15.72%. These data provide detailed information on the characteristics of AE reports related to Capmatinib, aiding in understanding the drug’s safety and related clinical outcomes.

**TABLE 3:** Basic information on AEs related to Capmatinib.

Factors	Number of events (%)
Gender	
Female	983 (49.37)
Male	791 (39.73)
Unknown	217 (10.90)
Age	
<18	2 (0.10)
18–45	12 (0.60)
45–65	109 (5.47)
65–75	214 (10.75)
≥75	242 (12.15)
Unknown	1412 (70.92)
Reporter	
Consumer	1067 (53.59)
Physician	582 (29.23)
Pharmacist	268 (13.46)
Unknown	74 (3.72)
Reported Countries	
United States	1245 (62.53)
France	86 (4.32)
Japan	41 (2.06)
Netherlands	31 (1.56)
Report Year	
2020	200 (10.05)
2021	603 (30.29)
2022	642 (32.25)
2023	546 (27.42)
Serious Outcomes	
Death	518 (26.02)
Disability	313 (15.72)
Hospitalization - Initial or Prolonged	36 (1.81)
Life-Threatening	29 (1.46)

### 3.2 AE signal mining results

The results of Capmatinib’s AE reports at the SOC level are presented in Table 4, involving a total of 26 SOCs, including gastrointestinal and various neurological disorders. Among these reports, the highest numbers were in General disorders and administration site conditions General disorders and administration site conditions (n = 1793, ROR 2.19 (2.07–2.32) PRR 1.81 (1.74–1.88), IC 0.85, EBGM 1.81), Gastrointestinal disorders (n = 615, ROR 1.48 (1.36–1.61), PRR 1.43 (1.32–1.54), IC 0.51, EBGM 1.42), Respiratory, thoracic and mediastinal disorders (n = 411, ROR 1.71 (1.55–1.89), PRR1.66 (1.51–1.82), IC0.73, EBGM 1.66), which are consistent with the drug’s labeling. Based on signal strength, the top three were Ear and labyrinth disorders (n = 60, ROR 2.67 (2.07–3.44), PRR 2.65 (2.06–3.41), IC 1.37, EBGM 2.65), General disorders and administration site conditions (n = 1793, ROR 2.19 (2.07–2.32), PRR1.81 (1.74–1.88), IC 0.85, EBGM 1.81), Hepatobiliary disorders (n = 94, ROR 2.11 (1.72–2.58), PRR 2.09 (1.71–2.55), IC 1.04, EBGM 2.09), with Ear and labyrinth disorders not listed in the label. Further comparison with the drug’s label revealed several other notable AEs that need attention in clinical practice, including Psychiatric disorders (n = 99, ROR 0.30 (0.25–0.37), PRR0.32 (0.26–0.38), IC -1.66, EBGM0.32)、Renal and urinary disorders (n = 94, ROR0.91 (0.74–1.11), PRR 0.91 (0.74–1.11), IC -0.14, EBGM0.91), Vascular disorders (n = 89, ROR 0.86 (0.70–1.06), PRR0.86 (0.70–1.06), IC-0.21, EBGM 0.86).

**TABLE 4:** The signal strength of AEs of Capmatinib at the SOC level.

System organ class	SOC code	Case reports	ROR (95% CI)	PRR (95% CI)	χ2	IC (IC025)	EBGM (EBGM05)
General disorders and administration site conditions	10018065	1793	2.19 (2.07–2.32)	1.81 (1.74–1.88)	787.18	0.85 (0.77)	1.81 (1.71)
Gastrointestinal disorders	10017947	615	1.48 (1.36–1.61)	1.43 (1.32–1.54)	84.46	0.51 (0.39)	1.42 (1.31)
Respiratory, thoracic and mediastinal disorders	10038738	411	1.71 (1.55–1.89)	1.66 (1.51–1.82)	112.72	0.73 (0.58)	1.66 (1.50)
Investigations	10022891	376	1.16 (1.04–1.29)	1.15 (1.04–1.27)	7.59	0.20 (0.04)	1.15 (1.03)
Nervous system disorders	10029205	264	0.64 (0.57–0.73)	0.66 (0.59–0.74)	50.45	−0.60 (−0.78)	0.66 (0.58)
Injury, poisoning and procedural complications	10022117	254	0.34 (0.30–0.39)	0.37 (0.33–0.42)	311.29	−1.43 (−1.62)	0.37 (0.33)
Musculoskeletal and connective tissue disorders	10028395	236	0.82 (0.72–0.94)	0.83 (0.73–0.94)	8.50	−0.27 (−0.46)	0.83 (0.73)
Metabolism and nutrition disorders	10027433	198	1.91 (1.66–2.21)	1.88 (1.64–2.16)	83.24	0.90 (0.70)	1.88 (1.63)
Skin and subcutaneous tissue disorders	10040785	170	0.57 (0.49–0.66)	0.58 (0.50–0.68)	53.21	−0.77 (−1.00)	0.58 (0.50)
Infections and infestations	10021881	141	0.44 (0.37–0.52)	0.45 (0.38–0.53)	99.35	−1.14 (−1.39)	0.45 (0.38)
Psychiatric disorders	10037175	99	0.30 (0.25–0.37)	0.32 (0.26–0.38)	156.25	−1.66 (−1.95)	0.32 (0.26)
Hepatobiliary disorders	10019805	94	2.11 (1.72–2.58)	2.09 (1.71–2.55)	53.68	1.04 (0.75)	2.09 (1.70)
Renal and urinary disorders	10038359	94	0.91 (0.74–1.11)	0.91 (0.74–1.11)	0.90	−0.14 (−0.44)	0.91 (0.74)
Vascular disorders	10047065	89	0.86 (0.70–1.06)	0.86 (0.70–1.06)	1.93	−0.21 (−0.52)	0.86 (0.70)
Ear and labyrinth disorders	10013993	60	2.67 (2.07–3.44)	2.65 (2.06–3.41)	61.85	1.37 (0.99)	2.65 (2.05)
Cardiac disorders	10007541	60	0.55 (0.42–0.71)	0.55 (0.43–0.71)	22.15	−0.84 (−1.22)	0.55 (0.43)
Product issues	10077536	44	0.42 (0.31–0.56)	0.42 (0.32–0.57)	34.98	−1.22 (−1.65)	0.42 (0.32)
Blood and lymphatic system disorders	10005329	41	0.43 (0.32–0.59)	0.44 (0.32–0.59)	30.39	−1.18 (−1.62)	0.44 (0.32)
Surgical and medical procedures	10042613	38	0.47 (0.34–0.64)	0.47 (0.34–0.64)	23.11	−1.07 (−1.53)	0.47 (0.34)
Eye disorders	10015919	34	0.31 (0.22–0.44)	0.32 (0.23–0.44)	50.66	−1.62 (−2.11)	0.32 (0.23)
Reproductive system and breast disorders	10038604	19	0.56 (0.36–0.89)	0.57 (0.36–0.89)	6.37	−0.79 (−1.44)	0.57 (0.36)
Immune system disorders	10021428	17	0.27 (0.17–0.44)	0.27 (0.17–0.44)	32.91	−1.80 (−2.49)	0.27 (0.17)
Congenital, familial and genetic disorders	10010331	5	0.37 (0.15–0.88)	0.37 (0.15–0.88)	5.43	−1.28 (−2.46)	0.37 (0.15)
Endocrine disorders	10,014,698	5	0.34 (0.14–0.81)	0.34 (0.14–0.82)	6.44	−1.39 (−2.57)	0.34 (0.14)
Social circumstances	10041244	2	0.07 (0.02–0.30)	0.07 (0.02–0.30)	23.06	−3.21 (−4.88)	0.07 (0.02)

After comprehensive evaluation, this study identified 269 PTs that met the criteria of all four algorithms. Following the most stringent EBGM algorithm analysis, these were ranked and the top 30 are displayed in Table 5. Notably, Peripheral swelling (n = 271, ROR 16.57 (14.66–18.73), PRR 15.81 (14.08–17.77), IC 3.90, EBGM 15.74)、Fatigue (n = 234, ROR 3.39 (2.97–3.86), PRR 3.29 (2.90–3.73), IC1.70, EBGM 3.29), Nausea (n = 219, ROR3.64 (3.18–4.17), PRR3.54 (3.11–4.03), IC 1.80, EBGM 3.53) had high signal frequencies, aligning with the drug’s label information. After excluding PTs unrelated to the drug (such as medical procedures, social environment, etc.), FDA-approved indications, and disease progression, the study also uncovered some noteworthy AE signals related to Capmatinib. Death (n = 377, ROR 4.96 (4.46–5.50), PRR4.69 (4.25–5.17), IC 2.21, EBGM 4.68) had the highest signal frequency; Vocal cord paralysis (n = 5, ROR38.71 (16.02–93.56), PRR 38.68 (16.02–93.41), IC2.41, EBGM 38.21) ranked second in signal strength; and Motion sickness (n = 3, ROR19.91 (6.40–61.98), PRR 19.90 (6.40–61.91), IC1.80, EBGM 19.78),Creatinine renal clearance decreased (n = 8, ROR27.61 (13.76–55.42), PRR 27.58 (13.75–55.29), IC2.80, EBGM 27.34, Amylase increased (n = 7, ROR23.42 (11.13–49.29), PRR23.39 (11.13–49.19), IC2.62, EBGM 23.22) were AEs that should be particularly monitored and treated with caution in the clinical use of the drug.

**TABLE 5:** The top signal strength of AEs of Capmatinib ranked by EBGM at the PTs level.

PTs	Case reports	ROR (95% CI)	PRR (95% CI)	χ2	IC (IC025)	EBGM (EBGM05)
Scrotal oedema	4	69.85 (25.92–188.25)	69.80 (25.92–187.99)	265.24	2.24 (0.93)	68.27 (25.33)
Vocal cord paralysis	5	38.71 (16.02–93.56)	38.68 (16.02–93.41)	181.25	2.41 (1.22)	38.21 (15.81)
Oedema	112	30.58 (25.34–36.90)	29.98 (24.94–36.05)	3109.74	4.57 (4.29)	29.70 (24.61)
Generalised oedema	25	29.76 (20.06–44.17)	29.63 (20.00–43.90)	685.18	3.81 (3.24)	29.36 (19.78)
Oedema peripheral	196	29.55 (25.61–34.10)	28.55 (24.86–32.78)	5168.99	4.64 (4.42)	28.30 (24.52)
Creatinine renal clearance decreased	8	27.61 (13.76–55.42)	27.58 (13.75–55.29)	203.08	2.80 (1.83)	27.34 (13.62)
Amylase increased	7	23.42 (11.13–49.29)	23.39 (11.13–49.19)	148.93	2.62 (1.59)	23.22 (11.04)
Motion sickness	3	19.91 (6.40–61.98)	19.90 (6.40–61.91)	53.51	1.80 (0.35)	19.78 (6.35)
Lymphoedema	12	17.97 (10.18–31.72)	17.93 (10.17–31.61)	190.79	2.96 (2.15)	17.84 (10.11)
Peripheral swelling	271	16.57 (14.66–18.73)	15.81 (14.08–17.77)	3752.98	3.90 (3.72)	15.74 (13.93)
Blood albumin decreased	7	15.08 (7.17–31.71)	15.07 (7.17–31.65)	91.48	2.45 (1.42)	15.00 (7.13)
Protein total decreased	3	13.56 (4.36–42.15)	13.55 (4.36–42.10)	34.72	1.71 (0.26)	13.49 (4.34)
Oesophageal stenosis	3	13.36 (4.30–41.54)	13.35 (4.30–41.49)	34.14	1.71 (0.26)	13.30 (4.28)
Product use complaint	26	12.92 (8.78–19.01)	12.87 (8.76–18.90)	283.48	3.16 (2.60)	12.82 (8.71)
Oesophageal pain	3	12.51 (4.02–38.88)	12.50 (4.02–38.84)	31.62	1.69 (0.24)	12.46 (4.01)
Gene mutation	3	11.65 (3.75–36.22)	11.65 (3.75–36.18)	29.09	1.67 (0.22)	11.61 (3.73)
Immune-mediated hepatitis	3	10.77 (3.47–33.47)	10.76 (3.47–33.43)	26.48	1.64 (0.20)	10.73 (3.45)
Blood creatine increased	4	10.22 (3.83–27.28)	10.21 (3.83–27.24)	33.13	1.84 (0.55)	10.18 (3.81)
Concomitant disease aggravated	6	10.09 (4.53–22.50)	10.08 (4.52–22.46)	48.92	2.13 (1.04)	10.05 (4.51)
Hypoalbuminaemia	6	9.91 (4.44–22.09)	9.90 (4.44–22.05)	47.84	2.12 (1.03)	9.87 (4.43)
Erysipelas	4	9.83 (3.68–26.25)	9.83 (3.68–26.22)	31.62	1.83 (0.53)	9.80 (3.67)
Hepatic cytolysis	17	9.34 (5.80–15.04)	9.31 (5.79–14.98)	125.81	2.67 (1.99)	9.29 (5.77)
Product supply issue	11	9.19 (5.08–16.61)	9.17 (5.08–16.56)	79.85	2.45 (1.61)	9.15 (5.06)
Fluid retention	36	8.97 (6.46–12.46)	8.92 (6.44–12.36)	252.66	2.87 (2.40)	8.90 (6.41)
Dysphagia	64	8.94 (6.98–11.44)	8.85 (6.93–11.29)	444.81	2.98 (2.62)	8.83 (6.90)
Blood creatinine increased	46	8.89 (6.65–11.88)	8.82 (6.61–11.77)	318.39	2.92 (2.49)	8.80 (6.58)
Hepatotoxicity	19	8.60 (5.48–13.50)	8.57 (5.47–13.44)	126.82	2.63 (1.99)	8.55 (5.45)
Pleurisy	3	8.30 (2.67–25.77)	8.29 (2.67–25.75)	19.19	1.55 (0.11)	8.27 (2.66)
Lipase increased	4	8.07 (3.02–21.53)	8.06 (3.02–21.50)	24.68	1.74 (0.45)	8.04 (3.01)
Deafness	18	7.64 (4.81–12.15)	7.62 (4.80–12.09)	103.33	2.50 (1.83)	7.60 (4.78)

## 4 Discussion

Capmatinib is a highly selective MET receptor inhibitor, exhibiting selectivity for MET over 1000 times greater than other targets (Hsu et al., 2023b). Mutations involving the skipping of MET exon 14 result in the loss of the regulatory domain of the protein, which diminishes its negative regulatory effects, thereby leading to increased downstream MET signaling. At appropriate concentrations, Capmatinib can inhibit the growth of cancer cells driven by MET mutants lacking exon 14, demonstrating its anti-tumor activity. Chemically, Capmatinib is known as 2-fluoro-N-methyl-4-[7-[(quinolin-6-yl) methyl] imidazo [1,2-b][1,2,4] triazin-2-yl] benzamide hydrochloride monohydrate. It belongs to a class of targeted kinase inhibitors that can inhibit a series of phosphorylation reactions associated with the MET activation pathway, including MET phosphorylation triggered by the binding of hepatocyte growth factor or MET amplification, as well as phosphorylation of downstream signaling proteins mediated by MET. It is particularly effective in treating non-small cell lung cancer (NSCLC) caused by MET exon 14 skipping (METex14) mutations. Importantly, direct alterations in the MET gene or activation of MET by HGF can confer resistance to most kinase inhibitors, leading to reduced drug efficacy and poor prognosis. However, Capmatinib can counter this resistance through its specific MET gene inhibition mechanism, significantly enhancing clinical outcomes (Baltschukat et al., 2019; Capmatinib, 2020). Due to its high efficacy and selectivity, it has been approved by the U.S. FDA for targeted treatment of NSCLC caused by specific genetic mutations (MET exon 14 skipping mutations).

As Capmatinib becomes more widely used in clinical practice, its safety issues have increasingly come into focus. Based on pharmacology and results from some clinical trials, there is now a clearer understanding of the potential adverse events caused by Capmatinib. In addition to common adverse reactions such as edema, fatigue, and nausea, the issue of Capmatinib toxicity has also become a focal point of concern. A clinical case report showed that two patients, without a history of primary liver disease, developed hepatocellular drug-induced liver injury (DILI) and exhibited elevated transaminase levels and early acute liver failure after being treated with Capmatinib. Combined with the FAERS drug vigilance analysis, there is a potential association between the use of Capmatinib and an increased susceptibility to developing DILI (Sisi et al., 2023). This study utilized the FAERS database, selecting 1,991 AE reports directly related to Capmatinib from the second quarter of 2020 to the fourth quarter of 2023, to analyze and evaluate potential AE signals of Capmatinib.

Numerous studies have confirmed that changes in c-MET (including amplification and mutation) positively correlate with increased expression of PD-L1 (Dempke and Fenchel, 2021). c-MET can regulate the immune response by upregulating inhibitory molecules (such as PD-L1) and downregulating immune-stimulating molecules, playing a regulatory role in the inflammatory tumor microenvironment (TME). As an inducible protein that inhibits innate and adaptive immunity, PD-L1 can develop a certain level of tolerance to recurrent infectious agents in the middle ear, reducing the extent of inflammation or immune response (Dong et al., 2002). Capmatinib, as a highly selective MET receptor inhibitor, might affect the expression of PD-L1 and inflammatory factors to varying degrees while blocking MET pathway activation. Therefore, the use of Capmatinib could decrease PD-L1 expression, weaken the tolerance to infectious agents mediated by it, and enhance the immune-inflammatory response, leading to ear diseases such as otitis media. Additionally, the outer wall fibrocytes of the cochlea have IL-10 α receptors, and IL-10 can activate these receptors and enter the cochlear internal environment, reducing the sensitivity of the vestibular cortex during unbalanced movements through a series of changes in the internal environment (Zhu et al., 2021). The use of Capmatinib might impact the secretion and expression of inflammatory factors (such as IL-10) through changes in c-MET, increasing the sensitivity of the vestibular cortex and potentially leading to motion sickness.

Significantly, AEs related to renal and urinary disorders were primarily characterized by renal impairment and decreased creatinine renal clearance. Several clinical case reports have indicated that the use of Capmatinib may induce pseudo-acute renal injury, mainly characterized by a close temporal association with increased serum creatinine levels (Mohan and Herrmann, 2022). The potential mechanism may be Capmatinib’s competitive inhibition of tubular creatinine transporters. Renal transport proteins, Multidrug and Toxin Extrusion Proteins 1 and 2-K (MATE1 and MATE2-K), and Organic Anion Transporter 2, primarily function in the secretion of a large amount of creatinine in the proximal tubules of the kidneys. Since creatinine is an endogenous substrate of renal transport proteins, the reversible increase in serum creatinine levels caused by Capmatinib might be due to its inhibition of the activity of the creatinine tubular transport proteins. This obstruction in the tubular transport and secretion of creatinine leads to increased serum creatinine levels. However, Capmatinib’s action targets the tubular transport proteins without affecting the activity of the glomeruli. Therefore, while serum creatinine levels rise, the glomerular filtration rate remains unchanged, resulting in pseudo-acute renal injury (Sandoval et al., 2023; Cui et al., 2023). It’s noteworthy that renal and urinary disorders induced by Capmatinib are currently characterized mainly by increased serum creatinine levels (pseudo-acute renal injury). Although there is no direct impact on renal function, the induced hypercreatininemia may affect the cardiovascular and skeletal systems of patients, thereby increasing the risk of other AEs.

## 5 Limitations of the study

Recent articles related to this study have focused on the characteristics of adverse event signals of Capmatinib in different genders, ages, weights, dosages, onset times, continents, concomitant medications, etc. This study confirms the association between objective factors such as gender, age, region, dosage, and adverse events of Capmatinib, and proposes reasonable hypotheses (Qi et al., 2024). At the same time, our study also confirmed hepatotoxicity caused by Capmatinib treatment and expanded the toxicity issues to include pancreatic toxicity and embryotoxicity. However, due to the inherent characteristics of the FAERS database, this study also has certain limitations. First, the database collects AE reports in a spontaneous reporting format, lacking strict supervision over patient personal information and actual drug use, potentially leading to underreporting, misreporting, or omission. Only a fraction of adverse events is typically reported to such databases, which means that the data may not fully represent the total incidence of adverse events related to a drug. Considering that many reports are submitted by consumers who may lack medical knowledge, there may be biases in the reported content of AEs, making it difficult to ascertain the accurate type and incidence of symptoms. Secondly, since Capmatinib was approved more recently, the number of AE report samples is relatively small, affecting the comprehensiveness and completeness of the results. Finally, this study is retrospective in nature; the statistical results only indicate a correlation between Capmatinib and specific AEs, not a definitive causal relationship. Additionally, since the FAERS database does not include information about patients’ existing diseases or concomitant medication use, these factors could influence the statistical analysis outcomes. Future research should include larger-scale controlled trials and clinical studies on Capmatinib to more comprehensively analyze its potential AEs and provide a safety assessment for its use.

## 6 Conclusion

This study conducted an in-depth analysis and evaluation of the potential AEs of Capmatinib using the FAERS database. The identified high-risk signals suggest that ear and labyrinth disorders and renal and urinary disorders require close attention in clinical use, with certain preventive measures needed to mitigate medication risks. Currently, the targeted therapeutic effects of Capmatinib are significant. In the future, molecular spectrum analysis diagnostics and combination therapies involving Capmatinib could lead to breakthrough progress in the treatment of non-small cell lung cancer. Furthermore, given the inherent limitations of the FAERS database, a more comprehensive safety assessment and deeper exploration of molecular mechanisms will be key research directions.

## Data Availability

The original contributions presented in the study are included in the article/supplementary material, further inquiries can be directed to the corresponding author.
